# Seeking Strategies to Optimize Blood Utilization: The Preliminary Experience with Implementing a Patient Blood Management Program in a Greek Tertiary Hospital

**DOI:** 10.3390/jcm10102141

**Published:** 2021-05-15

**Authors:** Aimilia Tsante, Anastasia Papandreadi, Andreas G. Tsantes, Elias Kyriakou, Panagiota Douramani, Electra Loukopoulou, Argyri Gialeraki, Styliani I. Kokoris, Athina Kypraiou, Aristarchos Poulis, Petros Kopterides, Daniele Piovani, Stefanos Bonovas, Serena Valsami, Argirios E. Tsantes

**Affiliations:** 1 Laboratory of Haematology and Blood Bank Unit, Attikon University Hospital, School of Medicine, National and Kapodistrian University of Athens, 12462 Athens, Greece; etsante@yahoo.gr (A.T.); andreas.tsantes@yahoo.com (A.G.T.); kyriakoue72@gmail.com (E.K.); akb@otenet.gr (P.D.); electra@hotmail.com (E.L.); agialer@med.uoa.gr (A.G.); stelkok19@gmail.com (S.I.K.); panagiotis1975@gmail.com (A.K.); a_poulis@otenet.gr (A.P.); 2 Information Technology Department, Attikon University Hospital, School of Medicine, National and Kapodistrian University of Athens, 12462 Athens, Greece; software@attikonhospital.gr; 3 Intensive Care Unit, Excela Health Westmoreland Hospital, Greensburg, PA 15601, USA; pkopterides@gmail.com; 4 Department of Biomedical Sciences, Humanitas University, Pieve Emanuele, 20090 Milan, Italy; 5 IRCCS Humanitas Research Hospital, Rozzano, 20089 Milan, Italy; 6 Department of Blood Transfusion, Aretaieion Hospital, School of Medicine, National and Kapodistrian University of Athens, 12462 Athens, Greece; serenavalsami@yahoo.com

**Keywords:** patient blood management, prospective audit, blood utilization, transfusion practice

## Abstract

Objectives: Our aim was to assess blood utilization after implementation of a patient blood management (PBM) program in a Greek tertiary hospital. Methods: An electronic transfusion request form and a prospective audit of transfusion practice were implemented. After the one-year implementation period, a retrospective review was performed to assess transfusion practice in medical patients. Results: Pre-PBM, a total of 9478 RBC units were transfused (mean: 1.75 units per patient) compared with 9289 transfused units (mean: 1.57 units per patient) post-PBM. Regarding the post-PBM period, the mean hemoglobin (Hb) level of the 3099 medical patients without comorbidities transfused was 7.19 ± 0.79 gr/dL. Among them, 2065 (66.6%) had Hb levels >7.0 gr/dL, while 167 (5.3%) had Hb levels >8.0 gr/dL. In addition, 331 (25.3%) of the transfused patients with comorbidities had Hb >8.0 gr/dL. The Hb transfusion thresholds significantly differed across the clinics (*p* < 0.001), while 21.8% of all medical non-bleeding patients received more than one RBC unit transfusion. Conclusion: A poor adherence with the restrictive transfusion threshold of 7.0 gr/dL was observed. The adoption of a less strict threshold might be a temporary step to allow physicians to become familiar with the program and be informed on the safety and advantages of the restrictive transfusion strategy.

## 1. Introduction

Red blood cell (RBC) transfusion is the mainstay to correct anemia, but it is also considered a frequently abused procedure since the indication for RBC transfusion remains uncertain. Some experts had previously predicted an impending blood shortage, mainly in industrialized countries, due to the increase in utilization from an aging population and a decrease in the percentage of people eligible to donate [1,2].

During the past decade, an increased emphasis has been given on optimizing blood utilization through a variety of modalities, including appropriate blood use through the successful implementation of evidence-based transfusion guidelines and highlighting the need to decrease unnecessary transfusions while ensuring that those who need blood get what they need. Patient blood management (PBM) has been defined as “the appropriate use of blood and blood components, with a goal of minimizing their use” [3] and refers to “the timely application of evidence-based medical and surgical concepts designed to maintain hemoglobin (Hb) concentration, optimize hemostasis and minimize blood loss in an effort to improve patient outcome” [4].

Auditing transfusion practice is considered a critical element of the process to optimize the use of blood products and should be a required function for all transfusion services. Audits combined with transfusion guidelines in association with real-time clinical decision support systems are considered critical [5].

According to data provided by the Greek National Blood Donation Center, Greece is one of the European Union countries with the largest collection of whole blood units and a higher rate of RBC transfusions per 1000 population [6]. In this context, many hospital blood banks have frequently experienced shortages of blood products. However, currently in Greece, there is no official requirement for a peer-reviewed program to monitor transfusion practices.

In 2019, in “Attikon” University Hospital, a tertiary medical center with 700 beds, about 10,000 RBC units were transfused while fewer than 9000 whole blood donations were collected on-site. Therefore, to close the gap between collections and demand, the hospital blood transfusion service developed and implemented, in collaboration with the Hospital Information Technology Department, a program to monitor and intervene in transfusion practices. It was against this backdrop that the transfusion medicine committee of “Attikon” University Hospital established local transfusion guidelines and implemented an electronic transfusion request system for in-patients. Local modifications of transfusion guidelines were made with the involvement of hospital stakeholders to increase adherence to guidelines. Then, a new computer order form for transfusions was introduced. Physicians had to check off at least one indication for each type of blood component order. Furthermore, laboratory and clinical information was required to perform the transfusion order. The data were transferred from the laboratory information system or obtained from the clinical staff as part of the transfusion request process. In this context, a prospective audit was applied, and the transfusion order was reviewed in real time and before issue of the blood components. Best practice alerts to educate the ordering physician were applied. In case of transfusions not meeting the audit criteria, the requesting physician was contacted by the senior technologist or attending physician before issuing the component to discuss the need for the transfusion.

A restrictive RBC transfusion threshold, in which the transfusion is not indicated until the hemoglobin level is 7.0 g/dL, was recommended for hospitalized adult patients who were hemodynamically stable, including critically ill patients. RBC transfusion threshold of 8.0 g/dL was recommended for patients undergoing orthopedic surgery, cardiac surgery, and those with preexisting cardiovascular or respiratory disease [7]. The same threshold was set for hematological/oncological patients. We also adopted the “transfuse and assess” strategy with each RBC unit [8].

Although PBM is considered a multidisciplinary approach, including a variety of modalities [9], our program included only the prospective audit of implementation of local transfusion guidelines, since its performance was a preliminary step. After a one-year implementation period, a retrospective review was performed to assess current transfusion practices regarding medical patients. Our aim was to draw conclusions in order to guide our long-term efforts to optimize the utilization of blood products.

## 2. Methods

The initial analysis examined the total number of RBC units transfused, the mean number of units transfused per patient, and the mean number of units transfused per hospitalized patient for a one-year period before and after implementing an electronic transfusion order system (from 1 October 2017 to 1 October 2018 and from 1 October 2019 to 1 October 2020, respectively). The time gap between the two study periods allowed physicians to be educated and become familiar with the new transfusion request form. To monitor transfusion practice in medical patients during the one-year prospective audit implementation period, additional analyses included estimation of the Hb thresholds used for RBC transfusions in clinical practice, proportion of inappropriate transfusions based on pre-specified audit criteria, and the mean number of RBC units transfused per transfusion episode or clinical specialty. A transfusion episode for medical patients was defined as each day a patient was transfused and the number of units per episode was calculated by adding all units transfused on the same date [8].

### Statistical Analysis

Statistical analysis included descriptive statistics of the study population for laboratory results and transfusion requirements. Data were presented as means ± standard deviations (SD), medians, and interquartile ranges (IQR) or percentages when appropriate. Hemoglobin transfusion thresholds were compared across hospital departments using the non-parametric Kruskal–Wallis test. The number of transfused RBC units was compared between oncology and non-oncology patients using the non-parametric Wilcoxon rank-sum test. Last, the percentages of transfused patients with different hemoglobin levels were compared between oncology and non-oncology patients using the chi-square test. We used STATA v15.0 (Stata Corp., College Station, TX, USA) for the statistical analysis.

## 3. Results

Pre-PBM, a total of 9478 RBC units were transfused (with a mean number of 1.75 units per transfused patient) compared with 9289 transfused post-PBM (with a mean number of 1.57 units per transfused patient) in the post-PBM period. The number of RBC units per hospitalized patient remained almost constant (0.25 and 0.26 in pre- and post-PBM era, respectively). Regarding medical patients during the post-PBM period, the mean Hb level of the 3099 medical patients without comorbidities transfused in our hospital was 7.19 ± 0.79 gr/dL, while the mean value of transfused RBC units per transfusion episode was 1.18 ± 0.42. The hemoglobin levels of those patients per hospital department are summarized in Table 1. The hemoglobin transfusion thresholds significantly differed across the departments (*p* < 0.001). Medical, hematology, and oncology patients were treated in the 2nd Department of internal medicine, while in the 4th Department, only medical patients were hospitalized. Hematologic diseases, malignancies, and gastrointestinal bleeding were among the most frequent indications for RBC transfusions. Among patients without comorbidities transfused in our hospital, 2065 (66.6%) had hemoglobin levels >7.0 gr/dL, while 167 (5.3%) had hemoglobin levels >8.0 gr/dL (Table 2).

Of the transfused patients without comorbidities, there were 1684 non-oncology patients with an average Hb level of 7.16 ± 0.86 gr/dL, while there were 1429 oncology patients with an average hemoglobin level of 7.24 ± 0.70 gr/dL. The percentage of transfused patients with hemoglobin levels >7.0 g/dL among the oncology patients was significantly higher compared to that of the non-oncology patients (*p* < 0.001).

Moreover, there were 1302 transfused patients with comorbidities with a mean Hb level of 7.62 ± 1.25 gr/dL (Table 3) who received on average 1.19 ± 0.49 RBC units per transfusion. Of the transfused patients with comorbidities, 331 (25.3%) had Hb level >8.0 gr/dL. In total, 21.8% of all medical non-bleeding patients received over one RBC unit transfusions.

## 4. Discussion

To evaluate current hospital transfusion practices, we initially observed a slight decrease in the total annual number of RBC units transfused pre-PBM as compared to post-PBM, while the mean number of units transfused per hospitalized patient remained almost unchanged. Because these data might display notable temporal variability, especially due to the emergence of the COVID-19 pandemic, the mean number of units per transfused patient provides a more meaningful summary of blood used in the post-PBM period compared to the pre-PBM one. A notable reduction of 10% in the number of units per transfused patient was observed after implementing the PBM program. However, we can only grossly compare the aforementioned parameters before and after the adoption of the new system, since the data obtained from the laboratory information system were cumulative rates and not individual patient data.

Estimating the current transfusion practice in medical patients, we observed a moderate compliance with the restrictive hemoglobin transfusion threshold of 7.0 g/dL in medical non-bleeding patients without comorbidities. A stronger adherence with the hemoglobin transfusion threshold of 8.0 g/dL in patients with or without comorbidities was detected. The threshold of 8.0 g/dL as a trigger for RBC transfusion was proved to be more easily adopted by physicians. By using the latter hemoglobin transfusion trigger, the proportion of inappropriate transfusions was notably lower than those reported in similar studies [10,11].

The current literature provides a scant rationale for selecting specific interventions, and there are few data to indicate which interventions are the most effective in changing transfusion practice. Contradictory findings are available regarding the impact of retrospective audits and feedback to clinicians on optimizing the use of blood products [12,13,14,15,16,17]. The prospective audit used to review transfusion requests before issuing components gives the means to intervene and stop or change an inappropriate transfusion request before the component is issued. The immediate intervention in a prospective audit could also lead to long-term changes in the transfusion practice of the ordering physician. Several single-center studies have shown that implementation of prospective audits resulted in lower consumption of blood components [18,19,20].

Our hospital transfusion service had to deal with a considerable gap between collections and utilization of blood components. To attain self-sufficiency in availability of blood components, implementing a prospective audit of transfusion practice was deemed as a necessary and reasonable option. Before that, the hospital transfusion committee, in collaboration with medical specialties that use high volumes of transfusions, established local transfusion guidelines to enhance acceptance of the audit criteria. Then, a two-month educational outreach program was designed and launched to inform, train, and increase engagement of physicians in implementing the new transfusion practice audit system. However, the poor participation levels of the physicians in the educational program was of great concern and led to significant inconvenience and friction during the introduction of the novel transfusion request form. A two-month trial period was applied for allowing the physicians to become familiar with the use of the new system, before its obligatory implementation. Pre-transfusion hemoglobin, comorbidities affecting oxygen carrying capacity such as coronary artery disease and unstable angina or myocardial infarction, hemodynamic instability, respiratory disease, presence of malignancy, presence of bleeding, and clinical indications for blood component transfusion were recorded. The appropriateness of transfusions was assessed based on both clinical and laboratory data supporting a “patient-based” transfusion practice. If parameters were not within recommended ranges and clinical data did not suggest the appropriateness of transfusion, the hospital transfusion service staff could intervene before the component issue and ask the requesting physician to explain the rationale of transfusing outside of the approved indications. However, individual review of transfusion requests proved to be a labor-intensive exercise for the inadequate transfusion service staff. Then, a one-year retrospective review period was defined, and the audit was performed to provide insight into aggregate transfusion data and trends in transfusion practice.

Considering hemoglobin level of 7.0 g/dL as the RBC transfusion threshold in medical non-bleeding patients without comorbidities, inappropriate transfusions took place in about two-thirds of patients. This rate significantly decreased to 5% of transfused patients if we were to accept a Hb level of 8.0 g/dL as the transfusion trigger, which is also a reasonable value adhering to a restrictive transfusion strategy (7.0 to 8.0 g/dL) in hospitalized, stable patients (Grade: strong recommendation; high-quality evidence) [21]; however, this was not the limit set in our hospital pre-specified transfusion criteria. Of note, significant differences in transfusion practice across several specialty groups were observed. Establishing hospital transfusion guidelines, we strongly encouraged the single-unit RBC transfusion strategy. Although a notable percentage of all medical non-bleeding patients received over one RBC unit transfusion, initial hemoglobin values and clinical needs had to be considered in the decision-making process for giving over one unit at a time.

The retrospective audit showed a rational use of blood components to a considerable extent. However, it is of note that no impressive differences were detected regarding parameters as the total number of RBCs transfused, the mean number of units transfused per patient, or the mean number of units transfused per hospitalized patient between pre- and post-PBM. These findings seem hard to explain at first sight. In Greece, a fully developed centralized blood supply chain network is not yet available. Thus, each hospital transfusion service has to ensure its self-sufficiency in blood components. Pre-PBM, “Attikon” Hospital Blood Bank Unit faced remarkable difficulties in collecting sufficient amounts of blood due to lack of adequate personnel and resources. However, despite the absence of both local transfusion guidelines and a peer-review program to monitor transfusion practice in the pre-PBM period, transfusion requests unavoidably came under scrutiny in order to bridge the gap between collections and demand. As a result, transfusion requests for patients with relatively high Hb values and non-accepted clinical indications had to be frequently rejected or postponed by the hospital transfusion service resulting in physicians’ animosity and suspiciousness, since audit criteria did not always seem objective and clear to them. Establishing local transfusion guidelines in collaboration with all involved physicians and implementing a prospective audit to review transfusion requests against pre-specified criteria was considered a more valid, transparent, and effective strategy to enhance optimal utilization of blood components.

In the post-PBM era, despite the use of such a prospective audit, a high rate of transfusions outside pre-specified local guidelines occurred. Several factors might account for the poor compliance with audit criteria. The reluctance of physicians to learn and adopt a new, labor intensive system of questionable value in their routine work was considerable. It is noteworthy that most physicians became familiar with the program only after its obligatory implementation by the transfusion medicine service. Despite the clear transfusion guidelines that had been developed by consensus, friction with requesting physicians was often observed when an “inappropriate” transfusion order was questioned. Since contact with the ordering physicians for all “inappropriate” transfusion requests needs additional work by the insufficient transfusion service staff, the sustained adherence to this intervention remains disputable. The occasionally intentional inclusion of false comorbidities in transfusion request forms by physicians in order to use the hemoglobin threshold of 8.0 g/dL as a transfusion trigger cannot be excluded. Given these conditions and considering the stronger compliance observed with hemoglobin transfusion triggers of 8.0 g/dL, audit criteria less stringent than optimal transfusion guidelines should be probably examined to decrease the audit workload and disputes with ordering physicians. However, in this case, current blood supplies also have to be taken under consideration before adopting a higher hemoglobin transfusion trigger.

Several limitations of the current study must be acknowledged. This is a single center study. Only a rough comparison between pre- and post-PBM period was feasible since limited data for evaluation of blood utilization were available pre-PBM. A more analytical insight into blood transfusion practice was obtainable only for the post-PBM era. Establishment of local transfusion guidelines and audit transfusion practice comprise just one component of a multidisciplinary, fully implemented PBM program. The study period was rather short to show the impact and the sustainability of PBM implementation on blood utilization. However, although limited, our data provide veritable insight into transfusion practices applied in a Greek tertiary hospital.

Our findings verified the concept that despite the benefits of PBM, its implementation is not an easy and convenient option because of several obstacles, including the commitment of time and staff, the unwillingness to adopt and develop new guidelines and procedures, and the lack of proper methods to monitor and show the effect of changes applied in PBM [22,23,24]. We experienced most of these barriers in our effort to implement our preliminary PBM plan. Of note, in our case, the reluctance of the local key stakeholders to be educated and properly prepared for implementing the new program, the questionable commitment of staff because of the consequent additional workload, and the adherence of physicians to ingrained institutional transfusion habits were the most prominent obstacles we had to overcome. Our aim was to move from a “product-based” transfusion practice to a “patient-based” one [25], by requiring providers to input transfusion indications at the time of order and consider factors beyond the hemoglobin level, such as diagnosis, comorbidities, and presence of bleeding. This strategy has proven helpful in carrying out personalized decisions for blood utilization and patient outcomes [26]. The tool we created not only required clinicians to provide a transfusion indication when ordering blood, but it also supplied the ordering provider with the patient’s most recent and relevant laboratory values and allowed deviation from the 7.0 g/dL transfusion threshold based on patient-specific symptoms and comorbidities. Despite all the aforementioned advantages, a poor adherence with the restrictive transfusion threshold was observed. Encouragingly, a significantly improved compliance was noticed considering a less stringent, but still acceptable, hemoglobin threshold. The adoption of a less strict threshold might be a temporary step in order that the physicians have the time to become familiar with the program and be informed on the safety and advantages of the restrictive transfusion strategy. However, avoidance of friction with clinicians should not be a priority and consequently impede or delay the process for establishment of PBM programs. Effective strategies for overwhelming the often deeply ingrained cultural and institutional barriers of behavior-based medicine should be designed and performed [27].

To achieve a broad implementation of PBM programs, establishment of national and institutional transfusion guidelines in collaboration with involved clinical specialties, obligatory stepwise performance of such programs, education on their potential advantages, and communication of post-PBM related metrics could be of critical value.

## Figures and Tables

**Table 1:** Hemoglobin levels of transfused patients without comorbidities.

Study Population	Hb (g/dL)
Total (*n* = 3099)	7.19 ± 0.79; 7.4 (6.8–7.7)
2nd Department of Internal Medicine (*n* = 1279)	7.16 ± 0.80; 7.4 (6.8–7.7)
2nd Department of Internal Medicine/Oncology (*n* = 303)	7.18 ± 0.69; 7.4 (6.9–7.7)
Department of Gastroenterology (*n* = 5)	7.08 ± 0.46; 6.9 (6.8–7.0)
4th Department of Internal Medicine (*n* = 311)	6.89 ± 1.01; 7.1 (6.4–7.6)
Critical Care Unit (*n* = 118)	7.08 ± 1.06; 7.0 (6.6–7.5)
Hematopoietic Stem Cell Transplantation unit (n = 164)	7.54 ± 0.59; 7.6 (7.3–7.9)
Department of Hematology (*n* = 903)	7.29 ± 0.64; 7.4 (7.0–7.7)
Department of Neurology (*n* = 1)	6.7
Emergency Department (*n* = 7)	7.71 ± 1.97; 7.6 (6.6–9.9)
Department of Dermatology (*n* = 4)	7.87 ± 0.20; 7.9 (7.75–8.0)
Department of Cardiology (*n* = 4)	8.27 ± 3.26; 7.65 (7.1–9.45)

Abbreviations: Hb, Hemoglobin; RBC, red blood cells. Data are presented as mean ± SD, median, and interquartile range (IQR).

**Table 2:** Transfused patients without comorbidities, and number of transfused RBC units.

Parameters	Total (*n* = 3099)
Patients with Hb > 7 g/dL	2065 (66.6)
Patients with Hb > 8 g/dL	167 (5.3)
RBC (units)/patient	1.18 ± 0.42; 1.0 (1.0–1.0)

Abbreviations: Hb, Hemoglobin; RBC, red blood cells. Data are presented as mean ± SD, median, and interquartile range (IQR), or as absolute value (percentage) when appropriate.

**Table 3:** Hemoglobin levels of transfused patients with comorbidities.

Study Population	Hb (g/dL)
Total (*n* = 1302)	7.62 ± 1.25; 7.5 (7.0–8.1)
2nd Department of Internal Medicine (*n* = 315)	7.37 ± 1.00; 7.5 (7.3–7.6)
2nd Department of Internal Medicine/Oncology (*n* = 187)	6.72 ± 0.39; 6.8 (6.4–7.1)
Department of Gastroenterology (*n* = 17)	7.70 ± 0.00; 7.7 (7.7–7.7)
4th Department of Internal Medicine (*n* = 512)	8.37 ± 1.44; 8.2 (7.85–8.90)
Critical Care Unit (*n* = 217)	6.94 ± 0.40; 7.1 (6.6–7.3)
Hematopoietic Stem Cell Transplantation unit (*n* = 5)	7.50 ± 0.00; 7.5 (7.5–7.5)
Emergency Department (*n* = 3)	7.53 ± 0.05; 7.5 (7.5–7.6)
Department of Dermatology (*n* = 1)	7.6
Department of Cardiology (*n* = 34)	7.81 ± 0.83; 7.9 (7.1–8.4)
Cardiac Care Unit (*n* = 11)	7.50 ± 0.00; 7.5 (7.5–7.5)

Abbreviations: Hb, Hemoglobin; RBC, red blood cells. Data are presented as mean ± SD, median, and interquartile range (IQR).

## Data Availability

The data presented in this study are available on request from the corresponding author.
